# Recommended Therapeutic INR Range for Patients with Antiphospholipid Syndrome on Warfarin Anticoagulation: Is Moderate-Intensity (INR 2.0 - 3.0) or High-Intensity (INR 3.1 - 4.0) Better for Reducing Risk of Recurrent Thromboembolic Events?

**DOI:** 10.7759/cureus.765

**Published:** 2016-09-01

**Authors:** Esther Kim, Tiffanie Do, Katie Peacock, Prisca T Takundwa

**Affiliations:** 1 College of Medicine, University of Central Florida

**Keywords:** antiphospholid syndrome, apl, inr, warfarin, thromboembolic

## Abstract

Patients with antiphospholipid syndrome (APS) are at increased risk of recurrent thromboembolic events due to the pathology of the disease. While prolonged anticoagulation is the treatment of choice for patients with thrombosis, much debate remains about the optimum intensity of anticoagulation. Anticoagulation with warfarin has been shown to decrease rates of thrombosis recurrence, but definitive evidence regarding targeted therapy to an INR of moderate (2.0 - 3.0) or high (3.1 - 4.0) intensity is lacking.

## Introduction and background

Antiphospholipid syndrome (APS) is an acquired systemic autoimmune disease characterized by thrombotic events, pregnancy complications, and the presence of antiphospholipid (aPL) antibodies. Diagnosis of aPL syndrome is based on the Sydney criteria, which is defined as either proven vascular thrombosis (venous, arterial, or small vessel) or pregnancy morbidity and the presence of aPL antibodies (anticardiolipin antibodies, beta2-glycoprotein antibodies, or lupus anticoagulant) on two or more occasions [1].

Studies have shown APS patients have more frequent thrombotic recurrences than non-APS patients [2-3]. Literature has supported positive aPL may correlate with thrombotic risk [4-5]. The thrombotic recurrence rates in APS patients have been suggested to range between one and 16/100 patients/year in various studies applying different classification criteria and therapeutic ranges [6-9]. It has been suggested the qualities of these studies are poor due to low adherence to classification criteria and standardization of laboratory tests [10]. Various studies have shown patients with APS require long-term anticoagulation with warfarin to reduce recurrent thromboses, morbidity, and mortality [2-3]. Previously, older retrospective studies suggested high-intensity anticoagulation to an INR > 3 in order to prevent recurrent thromboembolic events. More recently, however, two prospective, controlled therapeutic trials were unable to confirm the previous findings and demonstrated that lower intensity anticoagulation (INR: 2.0-3.0) sufficed in preventing recurrent thromboembolic events [6, 9].

A PubMed literature review was conducted to include articles published up to February 2016. Retrospective cohort and prospective randomized trials of patients with confirmed aPL syndrome and thrombotic events on anticoagulants were included. Case reports and case series were not included. Terms searched included antiphospholipid syndrome, recurrent thromboembolic, anticoagulation, and INR range. Data were extracted and analyzed to demonstrate that there exists an increased thrombotic risk for patients with aPL syndrome. Studies were reviewed to further elucidate appropriate anticoagulation intensity for APS patients.

## Review

Pengo, et al. [4] conducted a retrospective study that analyzed the clinical course of patients diagnosed with APS at four different time points; at diagnosis, after one year, five years and 10 years. The evaluated clinical outcomes include incidences of thromboembolic events (venous and arterial) as well as pregnancy morbidities. One hundred and sixty patients with a mean age of 41.1 +/- 15 years were enrolled in this study and qualified by having clinical features of APS in addition to being triple positive for APS, i.e. they had all three antiphospholipid (aPL) antibodies; lupus anticoagulant (LA), anticardiolipin (aCL), and anti-b2-Glycoprotein I (ab2GPI) antibodies. Patients were enrolled regardless of anticoagulation status. The thromboembolic events were defined as venous thromboembolisms (VTEs) at any site, intracerebral thrombosis, retinal thrombosis, peripheral or mesenteric thrombosis, acute myocardial infarction (MI), stroke, and transient ischemic attack (TIA) [4]. Pregnancy morbidity was defined as any pregnancy that was unsuccessful according to three set criteria: Type 1: fetus with normal morphology with unexplained death at or beyond 10 weeks gestational age; Type 2: eclampsia or severe eclampsia leading to pregnancy loss; and Type 3: three or more unexplained consecutive spontaneous abortions before 10 weeks gestation [4]. Major bleeding was also measured as another outcome.

The study showed that at the time of diagnosis, most patients with APS reported VTEs (47.5%), followed by arterial thromboembolism (43.1%) and pregnancy morbidity (6.9%) with the remainder having catastrophic events [4]. The collective incidences of events for the follow-up time points were 12.2% (95% CI, 9.6–14.8) after one year, 26.1% (95% CI, 22.3–29.9) after five years, and 44.2% (95% CI, 38.6–49.8) after 10 years. The study also demonstrated that the occurrence of outcomes in the successive time points was independent of the initial clinical presentation. From the 160 participants, 123 were on oral anticoagulation with a median INR target of 2.3. The remaining patients were either on aspirin or no therapy at all. A comparison of outcomes of these patients showed a higher incidence of thromboembolic events in the non-coagulated patients compared to those who were on coagulation therapy. In addition, patients on oral anticoagulation had fewer VTE events compared to arterial thromboembolic events. The only consistent prognosticator for whether or not thromboembolic events would occur at successive points was anticoagulation status. The authors of this study recommend anticoagulation therapy in all patients with VTE as well as arterial thromboembolic events (versus just antiplatelet therapy only for this group) with a target INR of 2.0 - 3.0 and increasing the target to 3.0 - 4.5 after a recurrence event.

Rosove, et al.’s retrospective study was conducted over three years to investigate the clinical course of patients with lupus anticoagulant and aCL antibodies [2]. Specifically, they looked at the effect of antithrombotic therapy on these patients. Seventy patients with a mean age of 45.5 +/- 17.3 years at secondary and tertiary centers were enrolled and were required to have had one thromboembolic event [2]. The patients in the study had a diagnosis of APS syndrome, double positive (anticoagulant and anticardiolipin antibodies positive) as a primary diagnosis or secondary to systemic lupus erythematosus (SLE) or chronic idiopathic thrombocytopenic purpura (ITP). The interventions included aspirin, heparin, and warfarin. Patients on warfarin therapy were stratified into low-intensity therapy (INR target < 1.9), intermediate intensity (INR 2.0 - 2.9), and high-intensity (INR > 3). Patients on heparin therapy were also stratified using different criteria. Clinical outcomes for thrombosis were defined as deep vein thrombosis; pulmonary embolism; cerebral vein/dural sinus thrombosis; renal, portal, or hepatic thrombosis; stroke; TIA; retinal artery thrombosis; amaurosis fugax; peripheral/mesentery artery thrombosis; spinal artery thrombosis, and acute MI [2]. The results showed that none of the enrolled patients were taking any anticoagulation therapy at the time of the first thromboembolic event. A 53% recurrence rate in thrombosis events was noted. This study observed that in these patients, venous events tend to recur as subsequent venous events and likewise with arterial events [2]. It is still possible to have an initial VTE followed by recurrences that are arterial in nature. In terms of anticoagulation interventions, the study showed that amongst the patients on warfarin therapy, the high-intensity group had the least number of recurrent thromboembolic events. Fifty-five patients were on warfarin with a total of nine recurrences in seven of these patients; six were in the low-intensity group, three in the intermediate group, and no recurrences in the high-intensity group [2]. Of note, five patients on warfarin therapy in the study experienced severe bleeding complications, including subdural hematomas. Two of these five patients had INR targets of 3 and 4 and at the time of these complications were found to have INR values of 6.4 and 26, respectively. From these observations, we can infer that while the high-intensity group in this study had no thromboembolism events, they did have some major bleeding complications. This bleeding risk, however, was shown to be comparable to the normal bleeding risk on warfarin therapy for all patients and not just APS patients. This study suggests that low-intensity therapy (INR < 1.9) does not offer adequate prophylaxis for recurrent thrombosis in APS patients [2]. For the remaining therapies in this study, aspirin was shown to offer no protection in APS patients and the results for patients on heparin therapy were inconclusive. This study recommends anticoagulation therapy with warfarin for APS with a target INR of 2.6 - 3.0 with adjustments as necessary according to perceived risk of bleeding [2]. The study was limited by its small sample size. Some of the patients in the study did not have measured INR targets and were assigned an arbitrary INR value of 2.6. Additionally, some of the patients on heparin therapy were excluded from the final results due to a high number of false positives in the group.

The Khamashta, et al. retrospective study assessed the efficacy of warfarin, aspirin, or both in secondary prevention of thrombosis in patients with antiphospholipid syndrome [3]. Over the course of a decade, 147 patients with antiphospholipid syndrome and a history of thrombosis were studied. All the patient data was collected from St. Thomas’ Hospital in the United Kingdom. All patients met the diagnostic criteria for antiphospholipid syndrome, namely, a positive laboratory test for antiphospholipid antibodies and a history of thrombosis (venous, arterial, or both) [3]. Patients with clinical manifestations of recurrent fetal loss were excluded. Of the 147 patients, 101 (69%) had a combined total of 186 thromboses. The retrospective analysis demonstrated that treatment with high-intensity warfarin (INR > 3) with or without low-dose aspirin (75 mg/day) was statistically effective at reducing recurrent thrombotic events when compared to low intensity (INR < 3) with or without low-dose aspirin or aspirin alone [3]. The study also emphasizes the need for anticoagulation by confirming other reports that patients with antiphospholipid syndrome are at an increased risk of recurrent thromboembolic events. The study limitations include the skewed population of women, with 84% of the enrollees identifying as female, as well as enrollment of patient data coming from a single hospital. Additionally, the retrospective design of the study limits the validity of the findings, and the authors encouraged further investigation on this topic.

More recently, Ruiz-Irastorza, et al. [11] conducted a retrospective cohort study of 66 patients in an attempt to clarify the risks and benefits of oral anticoagulation to a target INR of 3.5 in patients with diagnosed antiphospholipid syndrome and previous thrombosis. Patients were included in the cohort if they tested positive for APS on two separate occasions at least six weeks apart, had a history of thrombosis, and were treated with oral anticoagulants to a target INR of 3.5 during the previous 12 months [11]. Each patient was interviewed to recall specific bleeding episodes and thrombotic recurrences [11]. In order to reduce heterogeneity in treatments and anticoagulation surveillance as well as ensure more accurate recall from patients, this study was limited to the previous one year prior to the interview. Only episodes of major bleeding (intracranial, intraocular, gastrointestinal, retroperitoneal) or requirement of transfusion or hospital admission were included [11].

Results showed that the rate of major bleeding was six cases per 100 patient-years (95% CI, 1.6 - 15.0) while the rate of intracranial bleed was 1.5 per 100 patient-years (95% CI, 0.04 - 8.4) [11]. This study also found that most recurrent thrombotic events occurred in the same vasculature as the previous thrombosis. Additionally, the rate of thrombotic recurrences was 9.1 cases per 100 patient-years (95% CI, 3.3 - 19.6) [11]. Ruiz-Irastorza, et al. concluded that the risk of fatal or intracranial bleeding in patients with APS and previous thrombosis treated with oral anticoagulation to a target INR of 3.5 was similar to patient groups treated at lower target INRs [11]. The risk of thrombotic recurrences remained high despite anticoagulation.

This study was limited in several ways, most notably its retrospective design, which did not allow for proper documentation of the actual time at each range of INR. This is considered the most accurate method of monitoring treatment methods. Anticoagulation control was also not strictly adhered to as the INRs remained within the target range of 3.0 to 4.0 only 37% of the time [11]. Ruiz-Irastorza, et al. also had a limited sample population, which was predominantly Caucasian and had a female to male ratio of 9:1. This earlier study served to demonstrate the need for prospective randomized trials with a diverse patient population.

Crowther, et al. conducted the first randomized, double-blind trial to determine whether patients with aPL antibodies and previous thrombosis events would receive optimal treatment at an INR of 2.0 to 3.0 (moderate-intensity) or 3.1 to 4.0 (high-intensity) [6]. Since increasing the target INR from moderate to high-intensity is likely associated with doubling the risk of major hemorrhage, Crowther, et al. found importance in determining which was more effective. This investigation differed from previous studies as Crowther, et al. conducted a prospective trial that evaluated recurrent events objectively rather than relying on retrospective data on rates of recurrent thrombosis or anticoagulant intensity that could not be accurately determined.

Study patients were eligible if they met the following criteria: a positive test for aPL antibodies on two separate occasions at least three months apart and if they had confirmed arterial or venous thrombosis [6]. After screening 325 patients, 114 were enrolled in the trial. Most often, patients were excluded due to pregnancy, high risk of hemorrhage, or a previously failed attempt at moderate-intensity warfarin [6]. The average follow-up duration in the moderate and high-intensity group was 2.7 and 2.6 years, respectively. The moderate intensity group maintained an average INR value of 2.3 while the high-intensity group maintained an average INR value of 3.3. In the moderate-intensity group, the INR was within the range 71% of the time and was above and below range 11% and 19% of the time, respectively [6]. In the high-intensity group, the INR was within the range 40% of the time and was above and below range 17% and 43% of the time, respectively [6]. While the objective of Crowther, et al. was to show that high-intensity warfarin was more effective in preventing thrombosis than moderate-intensity warfarin, their data did not support the initial hypothesis. In this study, eight patients had recurrent thrombosis. Out of the 56 patients assigned to high-intensity therapy, six (10.7%) had recurrent thrombotic episodes, such as deep-vein thrombosis, stroke, pulmonary embolism, and myocardial infarctions. Their INR values also ranged from 0.9 to 3.9. Out of the 58 patients assigned moderate-intensity therapy, two had recurrences (3.4%) of deep-vein thrombosis and myocardial infarction, and their INRs had been 2.8 and 1.6, respectively [6]. Results showed that the absolute risk of recurrent thrombosis was low if the target INR of warfarin remained from 2.0 - 3.0.

This study had limitations in that there was a higher proportion of women in the moderate-intensity group by chance. The study also was unable to report on warfarin’s effectiveness in the initial three months after a first episode of thrombosis since the inclusion criteria required that patients have two positive antiphospholipid antibody tests separated by three months [6]. Additionally, results may have ultimately differed had the patients in the high-intensity warfarin therapy group remained within the target INR range for a larger portion of the study period.

The Warfarin in the AntiPhospholipid Syndrome (WAPS) study by Finazzi, et al. was the second randomized clinical trial to determine whether intensive anticoagulation was superior to the standard anticoagulation therapy in preventing thrombosis without increasing risk of bleeding in patients with antiphospholipid syndrome [9]. The single-blind study used 109 patients randomly assigned to either high (INR 3.1 - 4.0) or moderate/standard (INR 2.0 - 3.0) anticoagulation therapy [9]. The mean INRs for each group were within their targeted ranges and differed statistically. While the hypothesis of the trial was to demonstrate superior prevention in the high-intensity group based on previous retrospective studies, their results demonstrated more recurrent thrombosis in the high group compared to the low group, (11.1% vs 5.5%, respectively, hazard ratio 1.97, 95% CI, 0.49 - 7.89). Additionally, 15 patients (27.8%) in the high-intensity group compared to eight patients (14.6%) in the moderate-intensity group experienced major or minor bleeding, with statistical significance found when compared minor hemorrhage rates of high to moderate (27.8% in high vs 10.9% in moderate, HR 2.92, 95% CI, 1.13 - 7.52, p = 0.027) [9]. Their findings suggest that high-intensity warfarin treatment is not superior to moderate treatment in the prevention of recurrent thrombotic events and has an increased risk of bleeding when compared to moderate anticoagulation therapy.

The major limitations of both this clinical trial and the previous clinical trial [6, 9] are the small samples sizes and lack of statistical power due to the rarity of the disease and the low numbers of adverse events. However, before the control trials, anticoagulation therapy was primarily based on retrospective studies that reported definitely higher reduction rates when using higher intensity warfarin compared to moderate. The results from both clinical trials, while lacking in ideal statistical power, demonstrate the need for randomized control trials to assess the biologic theories put forth from retrospective studies.

Tan, et al. [12] conducted a retrospective study to investigate Singapore patients with APS that had events of venous and arterial thromboses. They speculated a target INR > 3 was associated with lower rates of recurrent arterial thromboses but higher rates of major and recurrent bleeding. Tan, et al. suggested a target INR ≥ 2 appeared to be sufficient to prevent events of recurrent venous thromboses. The study was limited because patients were not grouped according to predetermined target INR range; the mean INR for each patient was at times unable to be determined due to INR fluctuations, and INRs were not regulated to be tested at the same time interval for each and every patient. Moreover, the INR was recorded at the time when acute thrombosis was diagnosed. This recording was used as the patient’s INR at which thrombosis occurred. However, no studies have shown that a mean INR at an undefined time period before thrombosis is preferable to a single INR reading. As this study was a retrospective chart review, it suggests an association and not correlation.

The Piedmont cohort study by Bazzan, et al. was a multicenter observational study of 177 patients with vascular APS over a median course of five years [10]. This study analyzed the recurrence of thrombotic events while patients were on anticoagulant therapy. This study observed that 55% of patients were on anticoagulation therapy during the time of their first recurrent thrombotic event and about half of them were on oral anticoagulants targeted at INR 2.0 - 3.0 [10]. The limitations of this non-randomized cohort study included that the thrombotic recurrences were presumed to be related to early oral anticoagulant discontinuation or possible treatment failure. The study did not examine the INR at the specific time of thrombotic recurrence rendering the speculated INRs less accurate for possible association with treatment failure. With recurrent thrombotic events in patient INR levels of 2.0 – 3.0, the study suggested defining new anticoagulant strategies with higher INR in high-risk APL patients.

## Conclusions

While previous retrospective studies concluded that high-intensity anticoagulation therapy was more effective at thromboprophylaxis compared to moderate-intensity therapy, more recent prospective, randomized controlled trials have shown that there no longer remains a clear distinction between the two modalities of treatment. Additionally, though the prospective trials were randomized, controlled, and either single or double-blinded, they did not hold enough statistical power to make such definitive conclusions, which necessitates the need for further studies. This review of a series of studies over the past two decades demonstrates a possible shift in clinical management and treatment paradigms.

Additional prospective, randomized controlled studies are warranted with larger, diverse samples sizes and better adherence to target INR ranges. Also, many previous studies investigate preventing a majority of venous thromboembolic events; thus, we must also investigate more into prophylaxis against arterial thrombosis in patients with APS. This would elucidate the effect of proper anticoagulation therapy on APS patients to prevent recurrent thrombotic events. Future clinical trials will also help tailor treatment decisions to individual patients for improved clinical outcomes. 
